# Clinical Value of the Lactate/Albumin Ratio and Lactate/Albumin Ratio × Age Score in the Assessment of Prognosis in Patients With Sepsis

**DOI:** 10.3389/fmed.2021.732410

**Published:** 2021-10-15

**Authors:** Xiaonan Chen, Xinjian Zhou, Hui Zhao, Yanxue Wang, Hong Pan, Ke Ma, Zhijie Xia

**Affiliations:** Department of Emergency and Critical Care Medicine, Fudan University Affiliated North Huashan Hospital, Shanghai, China

**Keywords:** lactate, albumin, Lac/Alb ratio, sepsis, age score, disease prognosis assessment, clinical risk prediction

## Abstract

**Objective:** To examine the clinical significance of the blood lactate (Lac)/serum albumin (Alb) ratio and the Lac/Alb × age score for assessing the severity and prognosis of patients with sepsis.

**Methods:** A total of 8,029 patients with sepsis, aged >18 years were enrolled between June 2001 to October 2012 from the latest version of the Medical Information Mart for Intensive Care III (MIMIC-III v.1.4). The general data of the patients were obtained from hospital records and included gender, age, body mass index (BMI), laboratory indices, the sequential organ failure assessment (SOFA) score, and simplified acute physiology score II (SAPS II). The patients were graded and scored according to their age and then divided into a survival or death group based on their prognosis. The Lac/Alb ratio after ICU admission was calculated and compared between the two groups. The risk factors for death in patients with sepsis were determined using multivariate logistic regression analysis, while mortality was examined using receiver operating characteristic (ROC) curve and survival curve plots. Finally, the values of the Lac/Alb ratio and Lac/Alb × age score for assessing prognosis of patients with sepsis were analyzed and compared.

**Results:** After items with default values were excluded, a total of 4,555 patients with sepsis were enrolled (2,526 males and 2,029 females). 2,843 cases were classified as the death group and 1,712 cases in the survival group. (1) The mean age, BMI, SOFA and SAPS II scores were higher in the death group than those in the survival group. Significant differences in baseline data between the two groups were also observed. (2) The patients in the death group were divided further into four subgroups according to the quartile of the Lac/Alb ratio from low to high. Comparison of the four subgroups showed that the death rate rose with an increase in the Lac/Alb ratio, while analysis of the survival curve revealed that patients with a higher Lac/Alb ratio had a worse prognosis. (3) Multivariate logistic regression analysis showed that age ≥ 60 years, overweight (BMI ≥ 24 kg/m^2^), Lac/Alb ratio ≥ 0.16, SOFA score ≥ 2 points, and SAPS II ≥ 40 points were independent risk factors for death in patients with septic. (4) ROC curve analysis indicated that the SAPS II, Lac/Alb x age score, SOFA, and Lac/Alb ratio were the best predictors of death in patients with sepsis. The Lac/Alb × age score was characterized by its simple acquisition and ability to quickly analyze the prognosis of patients.

**Conclusion:** (1)A high Lac/Alb ratio is an independent risk factor for death in patients with sepsis. (2) Although the prognosis of sepsis can be accurately and comprehensively assessed by multi-dimensional analysis of multiple indices, **the Lac/Alb** **×** **age score is more accurate** and convenient for providing a general assessment of prognosis, so is worthy of further clinical recognition.

## Introduction

Sepsis is a clinical syndrome in which the host develops a systemic inflammatory response to infection (1) and life-threatening organ dysfunction (2) resulting in the condition being the major cause of death in critically ill patients. Although the understanding of sepsis has increased continuously and medical technology has improved rapidly in recent years, the death rate of patients with sepsis remains high due to the combined effects of disorders in circulation and cellular metabolism. It is, therefore, necessary to pay close attention to assess the outcome of the disease.

In addition to the early determination of the infection site and pathogen and aggressive fluid resuscitation, there is also a need to monitor various clinical indices to examine the therapeutic effect. Currently, blood lactate (Lac), an important parameter of tissue perfusion and infection, is used widely in clinical medicine. **Lactate can be increased significantly by** cellular ischemia and hypoxia and leads to metabolic disorders as a result of a further decline in the effective circulating volume of tissues. Hypoxia and energy failure are the primary conditions for the occurrence of an injury response, which may explain the adverse outcome of sepsis. A plasma albumin (Alb) level <35 g/L in adults indicates hypoalbuminemia, which often occurs concurrently in patients with sepsis, further worsening the disease and increasing the mortality rate. Many previous studies (1–3) have shown that a decrease in serum Alb level is an independent predictor of prognosis in patients with sepsis and septic shock. Therefore, blood Alb level is not only a nutritional index in patients, but also an important marker for the incidence of complications and mortality of patients with sepsis.

Based on the above premise, we consider that the Lac/Alb ratio may be a practical measure for assessing the severity of disease in patients with sepsis. We analyzed clinical information of patients with sepsis in the American critical care medicine information database (MIMIC-III v1.4), with the aim of determining the clinical significance of the Lac/Alb ratio alone or in combination with the age score for evaluating prognosis.

## Materials and Methods

### Data Source

The MIMIC-III database is an open intensive care medicine database jointly released by the Laboratory of Computational Physiology, Massachusetts Institute of Technology, Beth Israel Dikang Medical Center and Philips Healthcare under the funding of the National Institutes of Health. MIMIC-III v1.4 is the latest current version. Information on hospitalization of more than 50,000 patients admitted to the ICU of Beth Israel Dikang Medical Center from June 2001 to October 2012 was collected in the database (4), including vital signs, medications, laboratory measurements, observation results, records of nursing staff, fluid balance charts, program and diagnostic codes, imaging reports, length of stay, and survival data.

### Data Acquisition Process and Permission

After the Collaborative Institution Training Initiative (CITI) course was completed, access to the database was approved by the Review Committee of the affiliated institutions of Beth Israel Dikang Medical Center and the Massachusetts Institute of Technology. At the same time, the test for Protecting Human Research Participants was passed (Certificate No: 39691827), and the right to download and use the database was obtained.

### Inclusion and Exclusion Criteria

A total of 8,029 ICU patients aged >18 years and diagnosed with sepsis in accordance with the International Classification of Diseases (ICD-9) code 99591 and 99592 were included in the database. **Those with incomplete data were excluded**. For those with multiple records of hospitalization or multiple records of an ICU admission during the same hospitalization, only data from the first ICU admission during the first hospitalization were analyzed.

### Data Extraction

The following data were extracted from MIMIC-III using PostgreSQL 13 software and SQL: gender, age, body mass index (BMI), sequential organ failure assessment (SOFA) score, simplified acute physiology score II (SAPS II), white blood cell count, C-reactive protein (CRP), Lac and Alb levels, and prognostic indices. The patients were divided into a survival group or death group according to the presence or absence of an in-hospital death. Patients with incomplete data were deleted from the database. According to the age scoring rules for acute physiology and chronic health evaluation II (APACHE II), the patients were then further graded and scored based on age. As shown in Table 1, the range in scores according to age from young to old was 1–6 points. A check was carried out to determine whether Lac and Alb levels corresponded one to one with “charttime” to ensure that they were measured at the same time point. The Lac/Alb ratio was then calculated. For multiple Lac/Alb ratios measured after blood sampling in the same patient the mean value was used in the analyses.

**Table 1:** Age stratification and score of patients.

**Age**	≤44	45–54	55–64	65–74	≥74
**Score**	1	2	3	5	6

### Data Analysis

STATA 16.0 (Stata/MP for Windows, Version 16.0., StataCorp LLC, Texas, USA. Released 2019) was used for statistical analysis of the data. Continuous variables were subjected to the normality test and if they had a normal distribution they were expressed as mean ± standard deviation, with comparison between two groups carried out by independent-sample *t* tests. Variables with an non-normal distribution were expressed as median and quartiles and log-transformed for comparison of two groups using the Mann-Whitney U test. Comparison of nominal variables between two groups was carried out using the χ^2^ test. The risk factors for death in patients with sepsis were determined using multivariate logistic regression analysis and receiver operating characteristic (ROC) curve plots. The value of the Lac/Alb ratio, Lac/Alb × age score, Lac, CRP, SOFA score, and SAPS II for predicting prognosis in patients with sepsis was analyzed. The area under the ROC curve (AUC) was compared using the Z test. A *P*-value < 0.01 was considered to be statistically significant.

**Figure 1:**
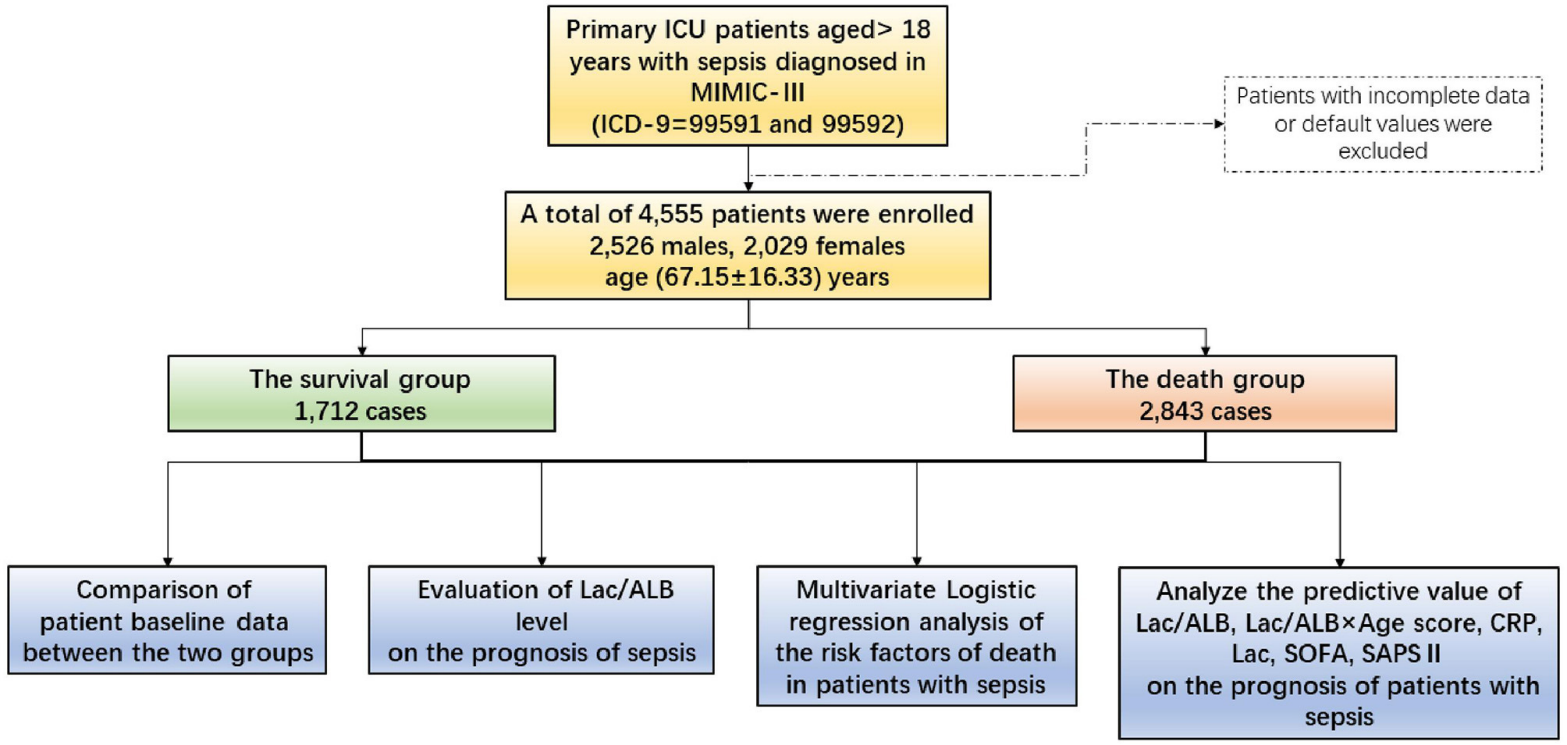
Graphical abstract of the study.

A summary of the statistical analyses is shown in Figure 1.

## Results

### Baseline Data of the Patients

**Figure 2:**
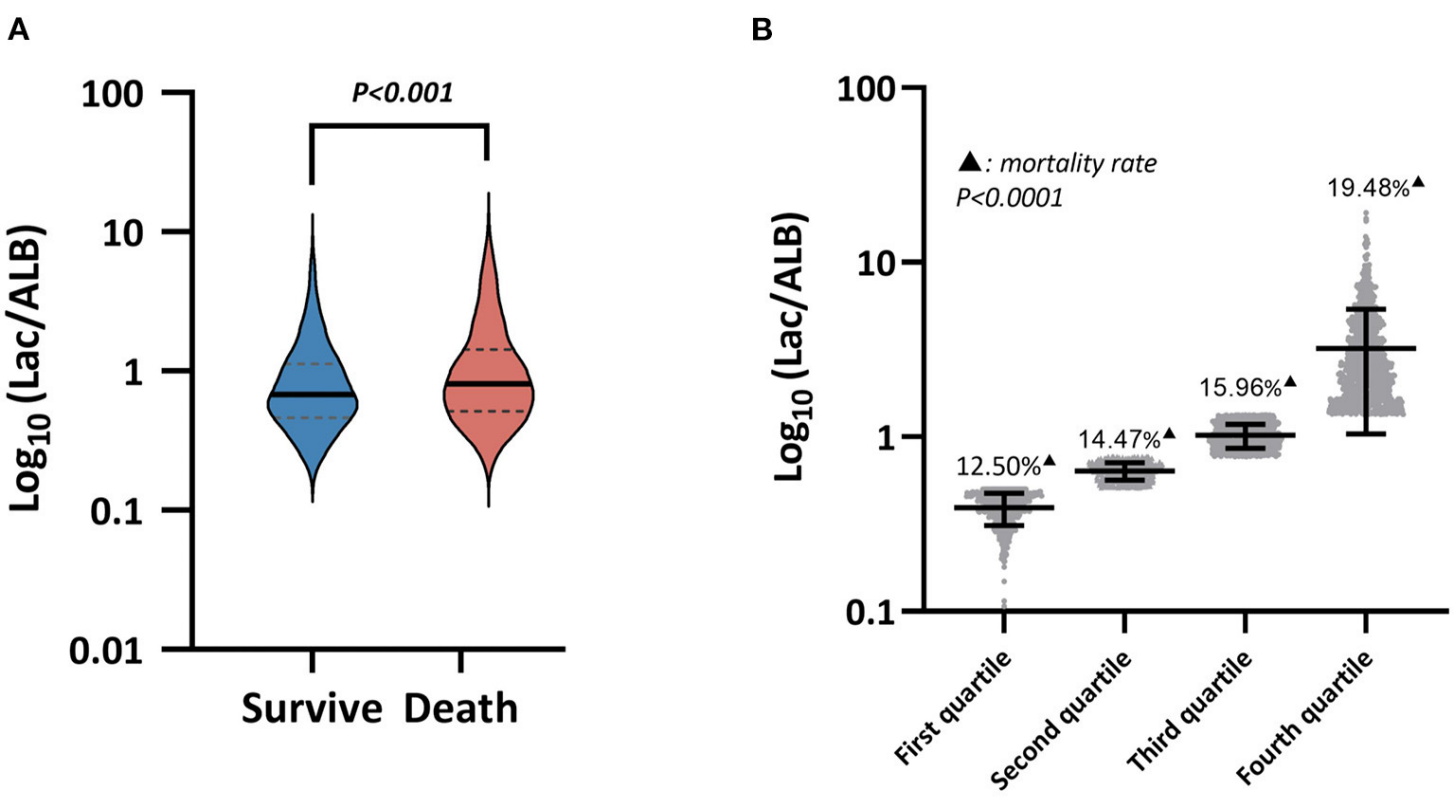
**(A)** Lac/A1b levels between the survival group and the death group, **(B)** Mortality among the four subgroups in the death group.

A total of 4,555 patients with sepsis were finally enrolled (2,526 males, 2,029 females), mean age 67.15 ± 16.33 years. The patients were divided into the survival or death groups according to the presence or absence of an in-hospital death. There were 2,843 cases in the death group and 1,712 cases in the survival group. The baseline data of patients are shown in Table 2. Compared to the survival group, the death group were older and had a higher BMI, while their SOFA score, SAPS II, and Lac/Alb ratio were all significantly increased (Table 2, Figure 2A). For vital signs, the death group had relatively lower body temperature and mean arterial pressure, and a higher mean heart rate and central venous pressure than that observed in the survival group. Organ function was also assessed in the two groups using the laboratory indices, with the results showing that cardiac and hepatic-renal function of patients in the death group was worse than that in the survival group (Table 2).

**Table 2:** Baseline data of patients.

**Characteristics**	**Total**	**Survival (*n = 1,712)***	**Death *(n = 2,843)***	***P-*value**
Age (Years)	67.15 ± 16.33	61.63 ± 17.00	70.46 ± 14.97	* <0.0001*
Male (*n*)	2,526	914	1,612	* <0.0001*
Female (*n*)	2,029	798	1,231	* <0.0001*
BMI	28.91 ± 8.12	28.24 ± 7.54	29.77 ± 8.73	* <0.0001*
SAPSII score	49.32 ± 16.81	42.33 ± 15.31	52.55 ± 16.49	* <0.0001*
SOFA score	8.34 ± 4.37	7.03 ± 3.95	8.95 ± 4.42	* <0.0001*
**Vital signs**				
Temperature (°C)	37.09 ± 1.02	37.36 ± 1.01	36.98 ± 1.01	* <0.0001*
Heart rate (BPM)	98.19 ± 14.76	92.47 ± 12.55	103.25 ± 18.03	* <0.0001*
MAP (mmHg)	78.51 ± 18.18	84.33 ± 18.45	76.61 ± 17.68	* <0.0001*
CVP (mmHg)	41.23 ± 86.81	23.88 ± 65.21	52.38 ± 96.58	* <0.0001*
**Laboratory tests**				
WBC (K/uL)	12.96 ± 9.04	12.92 ± 8.37	12.98 ± 9.37	* <0.0001*
N%	74.17 ± 21.76	76.37 ± 16.21	73.12 ± 23.90	* <0.0001*
Hemoglobin (g/dL)	9.70 ± 1.57	9.72 ± 1.63	9.69 ± 1.55	* <0.0001*
Platelet (K/uL)	212.32 ± 172.52	276.58 ± 199.32	179.65 ± 146.73	* <0.0001*
Na^+^ (mEq/L)	138.68 ± 5.70	139.05 ± 5.14	138.49 ± 5.96	* <0.0001*
K^+^ (mEq/L)	4.10 ± 0.66	4.03 ± 0.63	4.13 ± 0.67	* <0.0001*
HCO3^−^ (mEq/L)	23.84 ± 5.51	24.51 ± 5.15	23.50 ± 5.66	* <0.0001*
Cl^−^ (mEq/L)	104.43 ± 7.00	104.77 ± 6.51	104.25 ± 7.24	* <0.0001*
pH	7.36 ± 0.09	7.38 ± 0.09	7.36 ± 0.10	* <0.0001*
SaO_2_ (%)	88.53 ± 14.20	89.36 ± 12.84	88.23 ± 14.65	* <0.0001*
PO_2_ (mmHg)	115.41 ± 59.42	117.33 ± 58.19	114.60 ± 59.91	* <0.0001*
PaCO_2_ (mmHg)	41.56 ± 10.96	41.34 ± 9.44	41.65 ± 11.54	* <0.0001*
ALT (IU/L)	174.69 ± 587.46	146.80 ± 545.92	188.73 ± 606.85	* <0.0001*
AST (IU/L)	282.30 ± 1,119.73	189.74 ± 789.32	328.96 ± 1251.13	* <0.0001*
CRE (mg/dL)	1.81 ± 1.58	1.69 ± 1.66	1.87 ± 1.54	* <0.0001*
BUN (mg/dL)	40.11 ± 29.46	32.83 ± 25.95	43.91 ± 30.45	* <0.0001*
BNP (pg/mL)	9,305.18 ± 12,382.45	7,835.17 ± 10,794.02	9,855.29 ± 12,894.17	* <0.0001*
TnT (ng/mL)	0.56 ± 1.55	0.51 ± 1.07	0.57 ± 1.69	* <0.0001*
CK_MB (ng/mL)	14.69 ± 34.03	12.47 ± 29.91	15.76 ± 35.79	* <0.0001*
ALB (g/dL)	3.06 ± 3.12	3.28 ± 3.39	2.60 ± 2.41	* <0.0001*
Lactate (mmol/L)	2.69 ± 0.67	2.66 ± 0.66	2.76 ± 0.69	* <0.0001*

### Assessment of Prognosis of Patients With Sepsis by Lac/Alb

**Figure 3:**
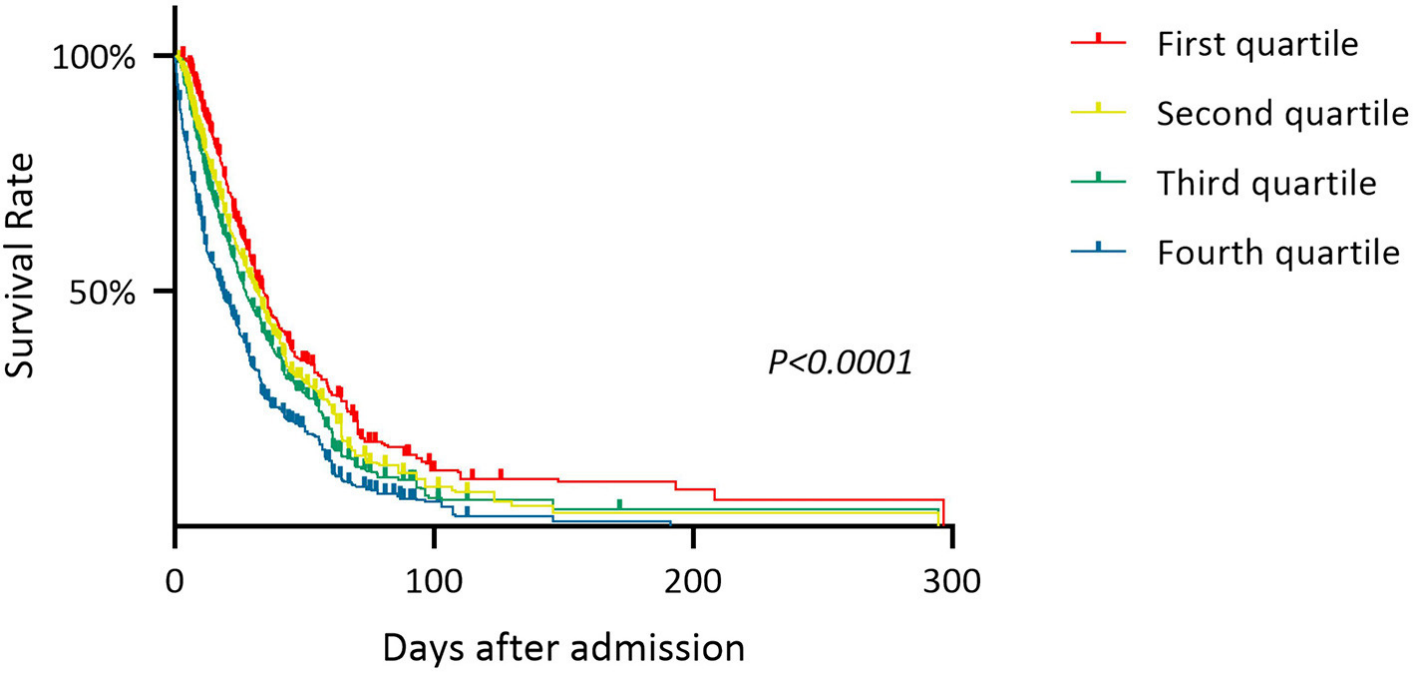
Overall survival expectations of sepsis patients with different Lac/A1b levels.

The patients in the death group were divided into four subgroups according to the quartile of the Lac/Alb ratio from high to low. The death rate was calculated as the ratio of the number of patients in the death subgroups relative to the total number of patients enrolled (Figure 2B). This showed that a higher Lac/Alb ratio was associated with a significantly higher death rate, with a significant difference observed between subgroups (*P* < *0.0001*). Long-term survival curve analysis also showed a close relationship between the Lac/Alb ratio and prognosis, with patients with a low ratio having longer survival (Figure 3). There was a significant difference in survival between subgroups (*P* < *0.0001*).

### Multivariate Logistic Regression Analysis of Risk Factors for Death

**Figure 4:**
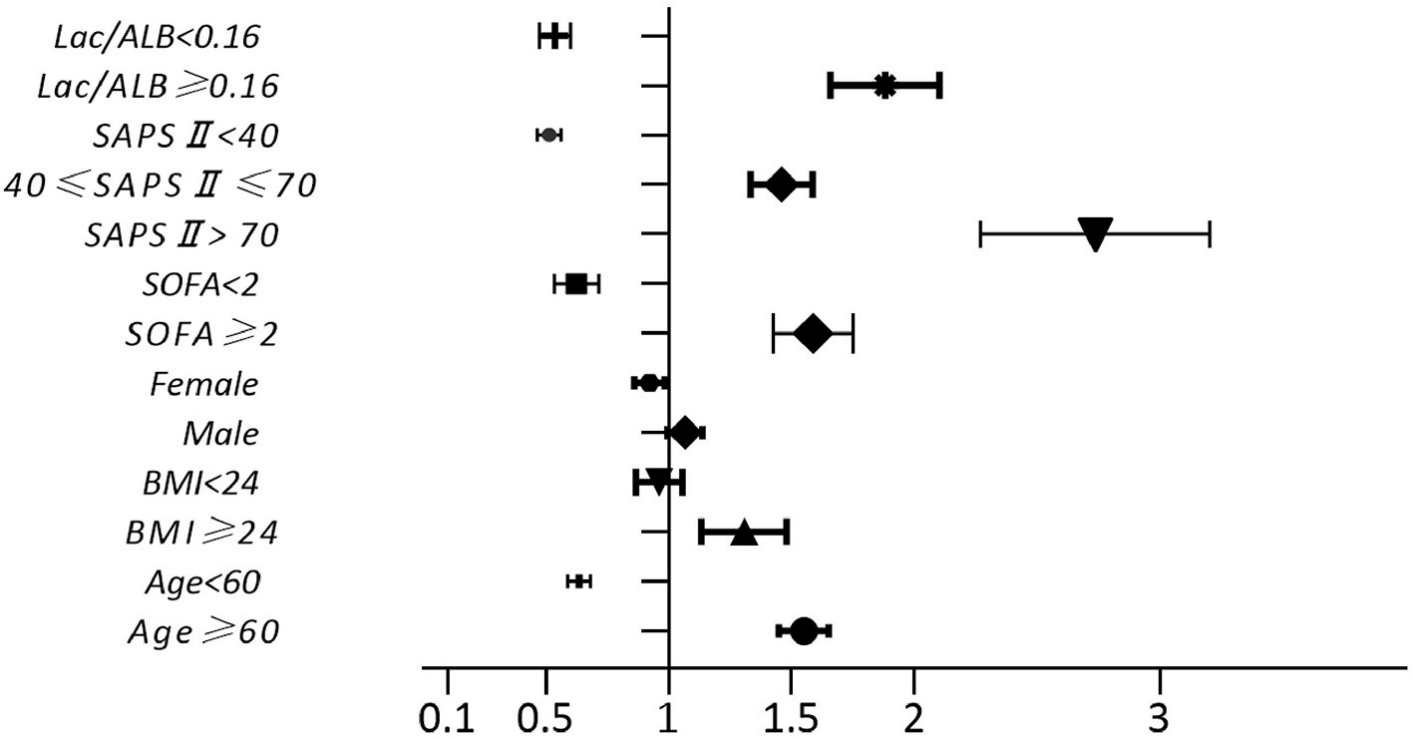
Mortality risk factors of patients with severe sepsis.

Multivariate logistic regression analysis was used to compare variables in the survival and death groups. This showed that age ≥ 60 years, BMI ≥ 24 kg/m^2^, SOFA score ≥ 2 points, Lac/Alb ratio ≥ 0.16 and SAPS II ≥ 40 points were independent risk factors for death in patients with sepsis (Figure 4, *P* < *0.001*).

### Predictive Value of Assessment Indices for Poor Prognosis in Patients With Sepsis

**Figure 5:**
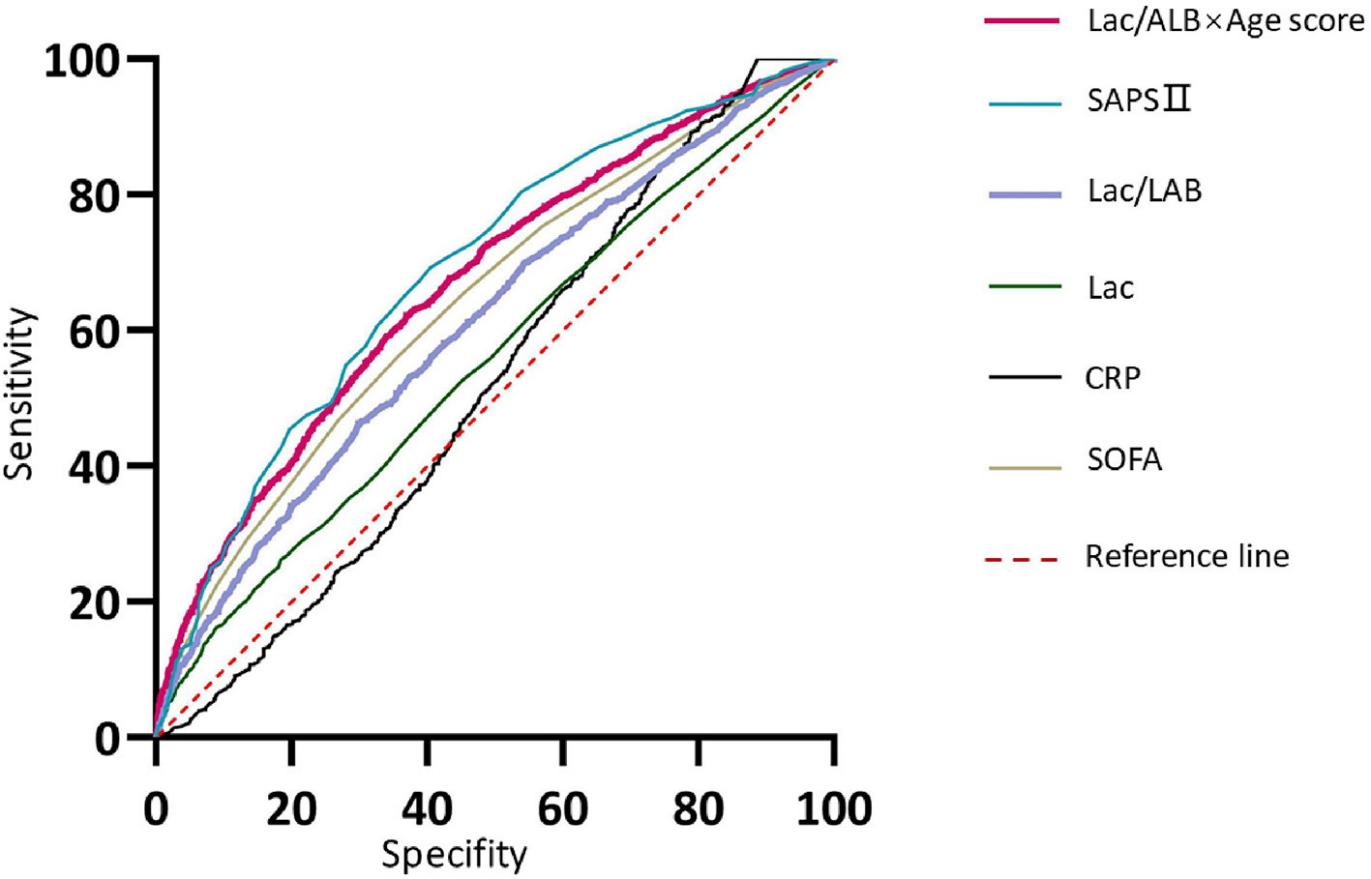
Prognostic value of indicators for predicting adverse outcomes in sepsis patients.

The value of the Lac/Alb ratio, Lac/Alb × age score, CRP, Lac, SOFA score, and SAPS II to predict prognosis in patients with sepsis was analyzed (Figure 5). The results showed that the predictive value of the Lac/Alb ratio and SOFA score were similar and not significantly different (Lac/Alb: AUC = 0.61, *P* < *0.0001*, 95%CI = 0.59–0.63, cut-off value = 0.16; SOFA: AUC = 0.64, *P* < *0.0001*, 95%CI = 0.63–0.66; AUC _Lac/Alb_ vs. AUC _SOFA_, *P* = *0.7384*). It was also found that the predictive value of the Lac/Alb × age score for sepsis (AUC = 0.67, *P* < *0.0001*, 95%CI = 0.65–0.68, cut-off value = 0.25) was better than that of the SOFA score, with a significant difference in AUC(AUC _Lac/Alb × *age*_ vs. AUC _SOFA_, *P* < *0.0001*). SAPS II had the highest predictive value for prognosis in patients with sepsis (AUC = 0.72, *P* < 0.0001, 95%CI = 0.70–0.73). Lactate had a relatively low predictive value (AUC = 0.56, *P* < *0.0001*, 95%CI = 0.54–0.57), while CRP had no predictive value (AUC = 0.52, *P* = *0.053*, 95%CI = 0.50–0.55).

The Z test was used to determine whether the predictive value of SAPS II and the Lac/Alb × age score was significantly different. The results showed no significant difference in the predictive potential of the two indices (*P* = *0.3266*).

## Discussion

Sepsis is the main cause of death in severely ill patients in ICU units. Recent studies in China and other countries have reported that the morbidity and mortality rates of sepsis remain high. These studies showed there are more than 30 million people in the world who suffer from sepsis every year, with any infected person possibly developing the condition The incidence rate of sepsis in hospital in-patients is 1–2% (5). Septic shock will develop in about 15% of these patients, occurring in about 10% of ICU patients, with a mortality rate above 50% (6). An epidemiological survey in China reported that the mortality rate of patients with sepsis was 48.7% (7), while a retrospective review of 419 patients with sepsis in a teaching hospital showed that the mortality rate of ICU patients was as high as 43.9% (8). These findings emphasize the importance of diagnosing and treating sepsis. Our results demonstrated that the in-hospital mortality rate in sepsis patients was as high as 62.4% with baseline data of the mortality group showing an association between death and older age, higher BMI, poorer organ function, and lower disease tolerance. Other factors possibly causing poor prognosis include more obvious progression of disease and greater organ dysfunction. Sepsis progresses very rapidly and its morbidity and mortality rates remain high despite good monitoring processes and diagnostic and treatment techniques, making it a major medical problem worldwide. In recent years, the guidelines on treatment for sepsis have been updated continuously, with the focus always on early identification of the pathogen and effective antibacterial medication. However, these etiological examinations are time-consuming and have a high false-negative rate, while the pathophysiological mechanism of sepsis is complex, involving immuno-inflammatory responses, cell function and metabolism, and blood coagulation abnormalities in the microcirculation. It is, therefore necessary for intervention strategies to combine anti-infection, organ protection, and fluid resuscitation. When changes in the condition of patients with sepsis are not assessed sufficiently early, the disease often leads rapidly to multiple organ and system failures, resulting in death.

Sepsis is a systemic inflammatory response syndrome caused by infection that is assessed usually by measuring body temperature, peripheral white blood cell count, percentage of neutrophils, CRP level, and organ function. However, these indices have very poor sensitivity and specificity, with our results confirming that these indices have low value for assessing the severity of sepsis and do not identify patients with a potentially poor prognosis. As a product of tissue anaerobic metabolism, Lac is an indicator of tissue hypoperfusion and cell hypoxia sensitivity, and also aerobic glycolysis, a key marker of the stress response and mitochondrial dysfunction. Coast et al. (9) showed that the Lac level in the early stage of trauma correlated with the severity of trauma and that an increase in level indicated an increased risk of mortality in patients, with a mortality rate of 0, 22, 78 and up to 100% when the level was lower than 1.4, 4.4, and 8.7 mmol/L and higher than 13 mmol/L, respectively. Clinically, blood Lac levels are usually monitored dynamically to assess the perfusion metabolism of tissue cells and the patient's response to treatment. During treatment of shock patients, the mortality rate will rise dramatically if their Lac level is high (10–12). The results of the current study showed that the Lac level in patients with sepsis was higher in the death group than that in survival group, suggesting that Lac levels reflect the severity of disease to some degree. In addition, Alb levels have a major effect on the maintenance of plasma colloid osmotic pressure. In an inflammatory storm, large quantities of inflammatory mediators are produced due to over-activation of the mononuclear phagocyte system, endothelial cells and neutrophils, that act on hepatocytes to inhibit expression of mRNA for Alb, triggering hypoproteinemia (13, 14). In addition, stress caused by severe infection accelerates the catabolism of serum albumin, significantly shortening its half-life. An increased distribution rate of Alb from intravascular to extravascular regions also reduces its serum levels (15). Our study, also found that Alb levels in the death group were markedly lower than those in the survival group, possibly as a consequence of its production and metabolism being adversely affected by other factors such as organ function and peripheral circulation. For example, clearance disorders and hepatic or renal insufficiency lead to abnormal levels of Lac and Alb. However, ROC curve analysis in our study showed that the Lac level alone could not be used to determine the severity or prognosis of the disease.

To help clinicians make treatment decisions for patients with sepsis, the severity or prognosis of the disease should be assessed as early as possible using multiple indices and prognostic scoring systems such as APACHE II, SOFA, and SAPS II (16, 17). APACHE II is used often to assess prognosis of respiratory, circulatory, and neurological diseases while SOFA provides an accurate assessment of the severity of sepsis and degree of organ damage. SAPS II is a modification of APACHE II but has fewer variables making it easier to collect. Recently, more attention has been paid to the value of the Lac/Alb ratio for assessing prognosis in critically ill patients as it reflects opposite changes caused by two different mechanisms with a normal or lower ratio indicating good prognosis. Shin et al. (18) carried out a multi-center retrospective study of patients with severe sepsis presenting to emergency departments of 10 teaching hospitals to evaluate the value of Lac/ALB to predict patient outcome and confirmed that the AUC of the Lac/Alb ratio was greater than that of Lac alone. Moustafa et al. (19) also studied pediatric patients with severe sepsis and found that the Lac/Alb ratio performed better than the Lac clearance rate for predicting the occurrence of the multiple organ dysfunction syndrome and death of patients. In addition, other studies confirmed that the Lac/Alb ratio is useful for risk stratification and predicting the risk of in-hospital death in patients with sepsis (20, 21). In early ICU hospitalization, the Lac/Alb ratio was also superior to APACHE II for predicting the development of MODS and mortality in septic patients (22, 23). The Lac/Alb ratio also plays a role in predicting the prognosis of other severe diseases. With similar Lac levels, the Lac/Alb ratio has been used to identify critically ill patients with heart failure (24), and as an early prognostic marker in ICU patients with different initial Lac levels or hepatic-renal insufficiency (25). Therefore, monitoring the Lac/Alb ratio may help to detect sepsis and initiate early treatment of critically ill patients.

The results of our detailed correlation analyses showed that an increase in the Lac/Alb ratio in patients with sepsis was associated with a gradual increase in the mortality rate and a corresponding decrease in survival rate. Because the levels of Lac and Alb show opposite changes with sepsis, the Lac/Alb ratio by integrating the two indices is able to sensitively reflect small changes in the condition of patients and therefore is an independent risk factor with good predictive potential for a poor prognosis. Because the MIMIC-III database did not contain the APACHE II scoring system data, SOFA and SAPS II were included as controls in the current study to assess the value of the Lac/Alb ratio for predicting the development of sepsis. The results showed that SAPS II had the strongest association with sepsis mortality, followed by SOFA and then the Lac/Alb ratio. These results indicate that although the Lac/Alb ratio can be used as an independent risk factor for death in patients with sepsis its predictive value is not greatly different from that of SOFA, and that it is best to use multiple indices for predicting a poor prognosis. Furthermore, when the Lac/Alb ratio was combined with the age score, the value of the Lac/Alb × age score to predict a poor prognosis from sepsis was further improved in that it was not only superior to SOFA but also comparable to SAPS II. The need for early detection and diagnosis of sepsis is the reason for carrying out these clinical measures, with a study reporting that treatment of a large number of patients with sepsis was delayed during an emergency due to failure of early identification and diagnosis, resulting in negative impact on prognosis (26). In clinical practice, indices that accurately assess the degree of sepsis and are easy and quick to acquire are extremely important. Several recent studies have compared different assessment modes and also developed new assessment tools for sepsis (27, 28) with the aim of obtaining a screening plan with good sensitivity and specificity. However, the effectiveness of these assessments remains to be validated by large-sample, multi-center studies. The SOFA score assesses six important system functions, while SAPS II evaluates 17 variables. Although multi-dimensional assessment integrating multiple variables greatly improves the predictive accuracy for sepsis, it is inconvenient to collect so many variables which is not conducive for early and rapid judgment of disease prognosis and treatment. The quick SOFA (qSOFA), a simpler scoring system, is therefore used commonly in clinics. This score incorporates systolic blood pressure, respiratory rate, and consciousness changes, making it easy to acquire and rapidly judge changes in a patient's condition. However, there is evidence that the specificity of qSOFA for assessing sepsis is unsatisfactory (29–31). The information of lactate and albumin is easy to obtain and can be reviewed in time with the changes of the disease. Lac/Alb × age score is also relatively accurate in the prediction of sepsis, which can be used as a convenient auxiliary means for early diagnosis and can also be used to closely evaluate the progress of sepsis.

## Limitations

Despite a large sample size and a long duration of retrospective data collection this study had some limitations. First, it was a single-center, retrospective study that limits the generalizability of the results. Large-sample multi-center prospective studies are therefore needed to validate the assessment potential of the indices. Second, no screening was carried out on underlying diseases that may possibly have affected the metabolism of Lac and Alb. Therefore, targeted studies on specific underlying disease groups are needed in the future. Finally, the APACHE II and qSOFA scoring systems and other prognostic indexes, such as procalcitonin used commonly to assess sepsis were not included in MIMIC-III. This may have caused certain deficiencies in the comparison and validation of the assessment ability of the Lac/Alb ratio and Lac/Alb × age score.

## Conclusion

In conclusion, the Lac/Alb ratio is an independent risk factor for death in patients with sepsis, and to a certain extent can be used to assess the severity of sepsis. Although the prognosis of sepsis can be assessed accurately and comprehensively by multi-dimensional analysis including multiple indexes, Lac/Alb × age score can give consideration to the accuracy and convenience of assessment to a certain extent, which has the value of further promotion in clinical practice.

## Data Availability Statement

The original contributions presented in the study are included in the article/supplementary material, further inquiries can be directed to the corresponding authors.

## Ethics Statement

All information related to the patients in MIMIC-III was anonymous, so informed consent was not needed. A review by the Ethics Committee of Huashan Hospital approved the study as it met all the conditions for exemption from review.

## Author Contributions

XC, HP, and HZ: conceptualization. XZ, YW, and KM: methodology. YW and XC: validation. XC: investigation and writing—original draft preparation. ZX: resources and funding acquisition. XZ and HZ: data curation. XC and HP: writing—review and editing. KM: project administration. All authors have read and agreed to the published version of the manuscript.

## Funding

This work was supported by: Special Project of Shanghai Municipal Economic and Information Technology Commission (Project No. 201601028), Key Medical Project of Science and Technology Support Plan of Shanghai Science and Technology Commission (Project No. 1641 1954400), and General Project of Shanghai Municipal Health and Family Planning Commission (Project No. 201640181).

## Conflict of Interest

The authors declare that the research was conducted in the absence of any commercial or financial relationships that could be construed as a potential conflict of interest.

## Publisher's Note

All claims expressed in this article are solely those of the authors and do not necessarily represent those of their affiliated organizations, or those of the publisher, the editors and the reviewers. Any product that may be evaluated in this article, or claim that may be made by its manufacturer, is not guaranteed or endorsed by the publisher.
